# Ascertaining Medication Use and Patient-Reported Outcomes via an App and Exploring Gamification in Patients With Multiple Sclerosis Treated With Interferon β-1b: Observational Study

**DOI:** 10.2196/31972

**Published:** 2022-03-14

**Authors:** Volker Limmroth, Kirsten Bayer-Gersmann, Christian Mueller, Markus Schürks

**Affiliations:** 1 Clinic for Neurology and Palliative Medicine Municipal Hospital Köln-Merheim Cologne Germany; 2 Institut Dr. Schauerte Munich Germany; 3 Bayer Vital GmbH Leverkusen Germany

**Keywords:** digital observational study, BETACONNECT, app, interferon β-1b, multiple sclerosis, medication adherence, medication compliance, medication persistence, health-related quality of life, gamification, mobile phone

## Abstract

**Background:**

The BETACONNECT autoinjector and myBETAapp app were designed to support patients with multiple sclerosis receiving interferon β-1b and are an ideal platform for digital observational studies. A recent pilot study in Germany demonstrated the feasibility of using the app to recruit patients, obtain informed consent, and evaluate medication-taking behavior over 6 months.

**Objective:**

This study aims to describe medication-taking behavior for 1 year in patients with multiple sclerosis receiving interferon β-1b based on data collected from the app and to provide information on patient-reported outcomes (PROs). The optional use of the cognitive training tool PEAK (Peak, formerly Brainbow Ltd) is included to test the feasibility of gamification in this setting.

**Methods:**

A prospective and retrospective, exploratory, digital, observational cohort study was conducted among users of the app in Germany. Invitations to participate were sent to patients’ apps between February and May 2019. Participants provided electronic informed consent. Injection-related data from consenting patients’ devices were collected prospectively for 1 year following the consent date and retrospectively for ≤1 year from the first day of use (if historical data were available). Participants also completed three electronic PRO instruments every 3 months: the EuroQol 5-Dimension, 5-Level questionnaire (EQ-5D-5L); the Treatment Satisfaction Questionnaire for Medication (TSQM; version II); and a questionnaire on satisfaction with treatment support (on a server accessed via an emailed hyperlink). All patients were offered optional access to the professional version of PEAK.

**Results:**

Of 1778 registered app accounts (May 2019), 79 patients (4.44%) provided informed consent; 62 (3.49%) were eligible for inclusion in the prospective analysis, of whom, 60 (97%) also had retrospective data. The mean age of the 62 participants was 43.2 (SD 11.5) years and 41 (66%) were women. Compliance over the 1-year prospective observational period (primary end point) was high (median 98.9%, IQR 94.3%-100%) and similar among men and women. Persistence and adherence (coprimary end points) decreased from 85% (53/62) and 74% (46/62), respectively, at 6 months to 76% (47/62) and 65% (40/62), respectively, at 12 months; both were higher in men than in women. A retrospective analysis showed similar patterns. The PRO questionnaires were answered by 79% (49/62) of the participants at baseline and 50% (31/62) of them at month 12. Women had more severe problems in some EQ-5D-5L dimensions (mobility, usual activities, and pain/discomfort) and lower median convenience scores on the TSQM (version II) than men. At month 12, 84% (26/31) of the patients were satisfied or very satisfied with the app. PEAK was used by 67% (14/21) of men and 49% (20/41) of women.

**Conclusions:**

This study showed high compliance and decreasing persistence and adherence over 1 year and demonstrated the feasibility of including remotely completed electronic PRO instruments in digital observational studies.

## Introduction

### Background

Digital clinical studies use technology to support recruitment and retention, data collection, and analytics [1], giving them several important advantages over conventional clinical studies. For example, slow recruitment is a common problem in clinical studies [2]. Digital studies allow rapid recruitment of patients outside of routine clinic visits (eg, by directly approaching them via their smartphones), with a corresponding reduction in study duration. In conventional clinical trials, the number of enrolled patients and number of clinic visits have been identified as key drivers of cost [3]. The inconvenience of making additional clinic visits is also a barrier to participation in clinical trials [4]. Digital studies avoid the need for clinic visits and allow enrollment of large numbers of patients at little extra cost once the digital study platform has been established.

In Germany, many patients with multiple sclerosis (MS) treated with interferon *β*-1b use the BETACONNECT autoinjector, which automatically records injections. An app (myBETAapp) allows patients who are being treated with interferon *β*-1b to document injection data manually or by automatic transfer from the autoinjector. This system is designed to support patients with MS, and it provides an ideal platform for digital observational studies. A recent pilot study in Germany demonstrated the feasibility of performing an observational study with digital recruitment, consent, and data collection performed via the app [5]. The pilot study results supported the extension of the digital observational approach to more comprehensive studies investigating clinical and patient-reported outcomes (PROs) over longer periods [5,6].

### Objectives

The aim of this digital observational study is to investigate medication-taking behavior in patients with MS receiving interferon *β*-1b for 1 year and to provide additional information on PROs. As cognitive impairment is common in MS [7], optional use of the cognitive training tool PEAK was included to test the feasibility of gamification in this setting and to incentivize patients to remain committed to the study.

Specifically, the primary objective is to describe medication-taking behavior prospectively for 1 year in patients with MS treated with interferon *β*-1b based on data collected from the app.

The secondary objectives are as follows: to investigate past medication-taking behavior (since first documentation in the app) before participation in the study; to evaluate health-related quality of life (HRQoL), treatment satisfaction, and satisfaction with treatment support prospectively for 1 year; to investigate HRQoL, treatment satisfaction, and satisfaction with treatment support at baseline in patients who were adherent or nonadherent as well as those who were persistent or nonpersistent over 1 year; to assess the relationship of HRQoL, treatment satisfaction, and satisfaction with treatment support at baseline with medication-taking behavior at 6 months and 1 year; and to evaluate how frequently and for how long the smartphone-based cognitive training tool PEAK was used.

## Methods

### Study Design and Patients

This was a prospective and retrospective, noninterventional, observational cohort study with the structure of a registry (ClinicalTrials.gov NCT03808142). Adult patients with MS treated with interferon *β*-1b, in Germany, were eligible to participate in the study if they were new or existing users of the app and provided electronic informed consent.

### Study Conduct and Ethics Approval

During a 3-month enrollment period (February 20, 2019, to May 19, 2019), all patients with an active account for the app were invited to participate in the study. After 2 months, invitations were sent to all new users of the app (patients who started using the app during the enrollment period). In both cases, the invitations consisted of push messages sent to the patients’ apps. Patients who did not respond to the initial invitation were sent a reminder after 2 weeks.

Patients who expressed interest in the study (by pressing a button within the app) were presented with a detailed informed consent form. The form provided study information in a sequential manner (background, aim of study, study design and data use, data privacy including how to withdraw consent, and contact information for the database hosts in case of questions), and the patients were required to confirm after each sequence that they understood the information and wished to participate. The form also clarified that prospectively recorded data and, among existing users of the app, injection-related data recorded in the past would be analyzed. It also included text to advise patients to report any side effects or possible side effects to their physicians, nurses, or Bayer, and to report any technical product complaints to Bayer. Only patients who consented to all the steps were able to participate in the study.

The study protocol was approved by the ethics committee of the Nordrhein Medical Chamber (number 2018381). In this observational study, interferon *β*-1b was prescribed in routine clinical practice by the treating physician in accordance with the terms of the marketing authorization, and the patient was offered support from the BETAPLUS Patient Support and Disease Management Program (PSDMP) as normal. The prescription of interferon *β*-1b and provision of the BETAPLUS PSDMP were clearly separated from the decision to include the patient in the study. No additional diagnostic or monitoring process was required for enrollment or during the study. Each patient could refuse to participate further or withdraw from the study at any time and without giving a reason. After withdrawal of the patient from the study, data from that patient were not used for further analyses. Each patient was assigned a unique central patient identification code, which was used only for study purposes.

### Data Collection

Demographic data (age and gender) were recorded in the app as part of the registration process. Injection-related data (date and time of injection, injection speed, and injection depth) were automatically recorded in the BETACONNECT for each injection and could be transferred to the app if the patient wished to do so. Patients could also manually record the injection data in the app. Demographic and injection-related data in the app were automatically transferred to an external database (hosted by TWT Digital Health GmbH) whenever the mobile device was connected to the web.

In addition, the patients completed three PRO instruments every 3 months from the time of informed consent to the end of observation: the EuroQol 5-Dimension, 5-Level questionnaire (EQ-5D-5L); the Treatment Satisfaction Questionnaire for Medication (TSQM; version II) [8,9]; and a questionnaire on satisfaction with the BETAPLUS PSDMP, the BETACONNECT, and the app (service questionnaire; Multimedia Appendix 1). For this purpose, each patient was provided with an individual log-in hyperlink (newly created for each data capture time point) via email to enter an electronic data capture server hosted by the contract research organization, Institut Dr. Schauerte (IDS). Hence, the participants were able to complete the questionnaires remotely. After the end of the observation period, participants received vouchers for completing each questionnaire.

**Figure 1:**
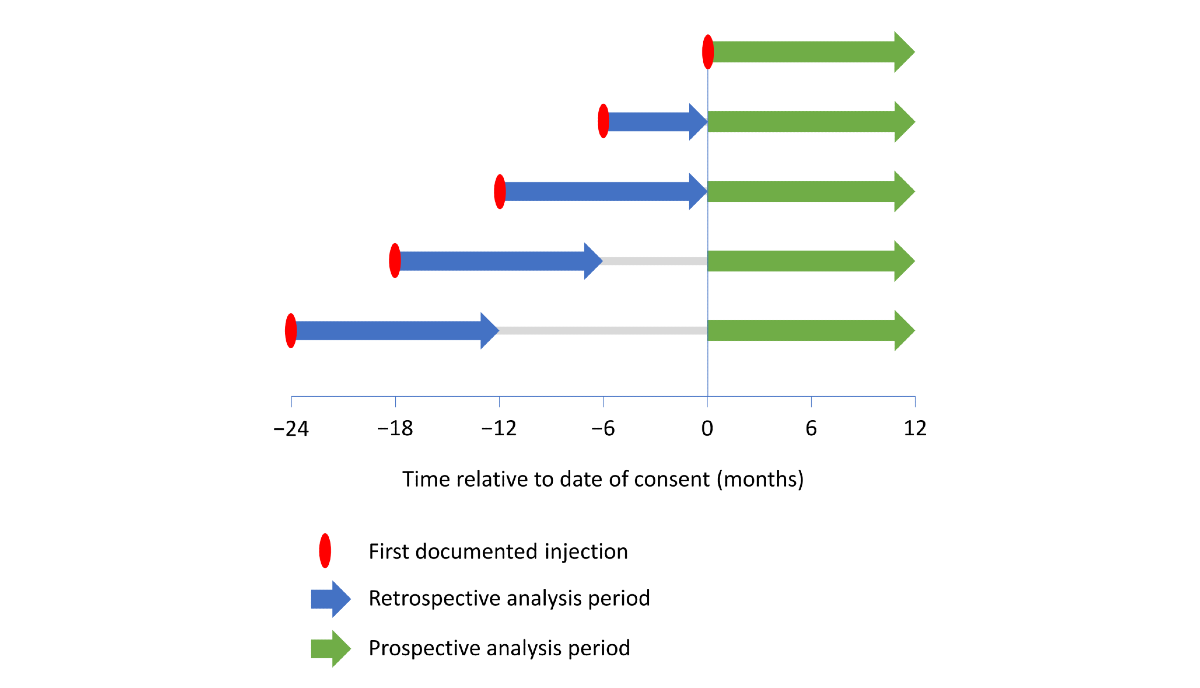
Examples of retrospective and prospective analysis periods for patients with a first documented injection at different intervals before the date of consent.

Each patient was followed up for 1 year after providing informed consent (prospective analysis). For patients who had already started using the app before providing informed consent, prospectively collected injection-related data (BETACONNECT or manual records) were also available from the past. Among those patients, medication-taking behavior was also analyzed from the time of the first documented injection to 1 year later or the date of consent, whichever was earlier (retrospective analysis; Figure 1).

All patients were initially offered access to the professional version of the mobile-based cognitive training tool PEAK, developed and hosted by Peak (formerly Brainbow Ltd); this offer was renewed with each request to complete the PRO questionnaires (quarterly emailing of log-in hyperlinks). The patients were able to choose from all available games within PEAK, covering 7 cognitive areas, for the duration of the study. The decision to use the training tool and game selection was purely voluntary and was not linked to treatment, study participation, or completion of the PRO instruments. All data generated by the patients while using PEAK were stored in a database hosted by Peak. The patients received study-specific log-in data, which identified them as participants of the study in the database, without personal identification.

Log-in details for the questionnaires on the IDS electronic data capture server and for PEAK were allocated to participants by TWT Digital Health GmbH. Hence, only TWT Digital Health GmbH had the key to match data from Peak, IDS, and their own database. Data sets coded by log-in identifiers were transferred from Peak and TWT Digital Health GmbH to IDS, who merged these into their own data set. No investigator was involved in the data collection process.

Before the analysis, redundancies in the injection data were automatically corrected as described previously [5].

### Outcome Measures

The primary end point was prospectively assessed compliance (the percentage of expected doses actually injected), based on injections recorded in the TWT Digital Health GmbH database from the date of informed consent to the end of the observation period.

The coprimary end points were persistence (the number of patients still using interferon *β*-1b at the end of the observation period), missed injections (expressed as a proportion of expected injections), and adherence (the number of patients who were both persistent and ≥80% compliant; a threshold of 80% is commonly used in studies of adherence in MS [10]). Patients without injection recordings for >2 weeks were regarded as nonpersistent.

Compliance, persistence, missed injections, and adherence were also analyzed retrospectively (from the first injection recorded in the app until 1 year later or the date of informed consent, whichever was earlier) as secondary end points among participants who had registered and used the app before enrolling in this study.

Other secondary end points included HRQoL assessed using the EQ-5D-5L, treatment satisfaction assessed using the TSQM (version II) [8,9], satisfaction with treatment support assessed using the service questionnaire (Multimedia Appendix 1), and use of the PEAK cognitive training tool. Frequency (trainings/week) and duration (days) of PEAK use by study participants and cumulative time played per game (minutes) were analyzed. For the analysis of the frequency of PEAK use and cumulative time played per game, only the 10 most frequently used games were considered.

### Statistical Analysis

Statistical analyses were exploratory and descriptive, using summary statistics for categorical and quantitative (continuous) data. Associations of persistence and adherence with baseline HRQoL, treatment satisfaction, and satisfaction with support were evaluated using univariate logistic regression analysis, with *P*<.05 considered significant. All statistical analyses were performed using the software package SAS 9.4 (SAS Institute Inc).

Assuming an estimated SD of 15 for the mean compliance (as observed in the pilot study [5]), a sample size of 100 would produce a 2-sided 95% CI with a half-width of 3.0.

## Results

### Participants

Of the 1778 registered accounts for the app (May 2019), 79 (4.44%) patients provided informed consent to participate in the study. Of the 79 patients, 10 (13%) patients withdrew their consent, 6 (8%) patients were excluded because they were enrolled after the end of the registration period, and 1 (1%) patient had no injection or questionnaire data. Thus, 78% (62/79) of patients had injection-related data and were valid for inclusion in the prospective analysis. Of the 62 patients, 2 (3%) had no retrospective injection data and were therefore excluded from the retrospective analysis.

The mean age of the 62 participants was 43.2 (SD 11.5) years; 41 (66%) were women, and 21 (34%) were men. The distribution of age groups in women and men is presented in Table 1.

**Table 1:** Study participants according to age group and gender.

Age group (years)	Participants, n (%)		
	Total (N=62)	Female (n=41)	Male (n=21)
<30	9 (15)	8 (20)	1 (5)
30 to <40	15 (24)	9 (22)	6 (29)
40 to <50	19 (31)	11 (27)	8 (38)
50 to <60	13 (21)	9 (22)	4 (19)
≥60	6 (10)	4 (10)	2 (10)

### Data Collected

Of the 62 patients, 14 (23%) used the BETACONNECT autoinjector to administer and record injections throughout the prospective observation period. For 19% (12/62) of the patients, only manual records of injections were available. Alternating recording methods (autoinjector or manual) were documented in 56% (35/62) of the patients. Switching from one mode of injection to the other (autoinjector to manual or vice versa) was documented in 2% (1/62) of the patients.

Use of the BETACONNECT to record injections was more common among men (8/21, 38%) than among women (6/41, 15%), whereas only manual recording of injections was more common among women (10/41, 24%) than among men (2/21, 10%). The injection location data were recorded for all 62 patients.

For the prospective analysis (N=62), 7 (11%) patients had <6 months of injection data and 55 (89%) patients had ≥6 months of injection data, with 48 of the 55 patients (87%) having ≥12 months of injection data. For the retrospective analysis (n=60), 8 (13%), 52 (87%), and 44 (73%) patients had <6, ≥6, and ≥12 months of injection data, respectively.

Of the 62 patients, 53 (85%) answered at least one questionnaire, and 34 (55%) used PEAK at least once. The EQ-5D-5L, TSQM (version II), and service questionnaire were each answered by 49 (79%) of the 62 patients at baseline and by 36 (58%), 35 (56%), 32 (52%), and 31 (50%) patients at months 3, 6, 9, and 12, respectively.

### Medication-Taking Behavior

**Figure 2:**
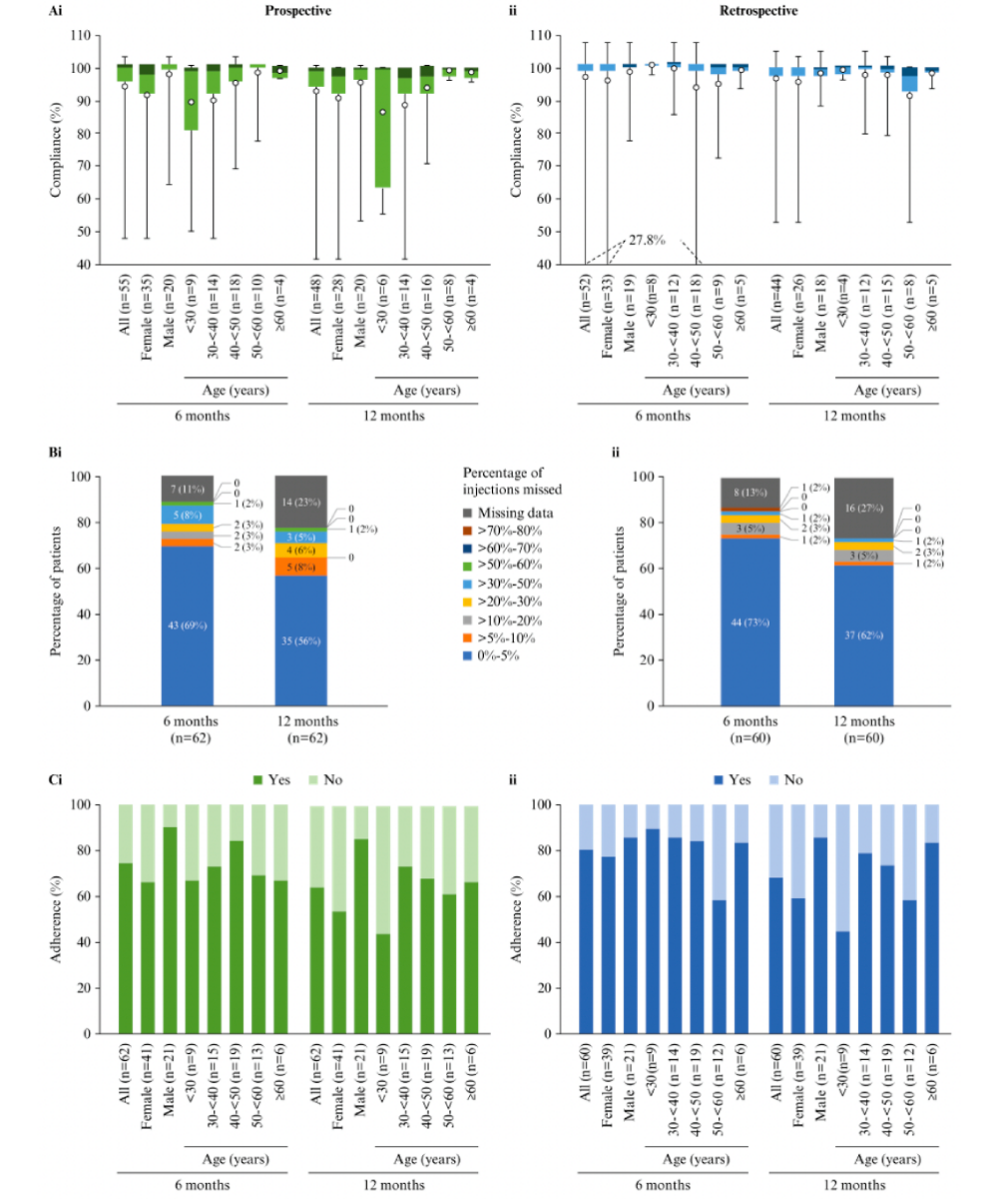
(A) Compliance overall and by gender and age group, (B) patients categorized by percentage of interferon β-1b injections missed (based on an expected frequency of 1 injection every other day), and (C) adherence overall and by gender and age group, analyzed (i) prospectively and (ii) retrospectively. Compliance at 6 and 12 months was assessed in patients with ≥6 and ≥12 months of injection-related data, respectively. In the box and whisker plots, the colored bars indicate median and IQR, the whiskers indicate minimum and maximum values, and the white circles indicate the mean.

In the prospective analysis, persistence was 85% (53/62) at 6 months and decreased to 76% (47/62) at 12 months after the date of informed consent (Table 2). Persistence was higher in men than in women at both 6 months (20/21, 95% vs 33/41, 80%) and 12 months (19/21, 90% vs 28/41, 68%; Table 2). Compliance (the primary end point, based on injection data up to 6 months and up to 12 months in patients with injection data available for those time periods) was high at 6 months (mean 93.6%, SD 13.8%; median 100%, IQR 95.6%-101.1%) and at 12 months (mean 92.6%, SD 14.1%; median 98.9%, IQR 94.3%-100%), and was similar in men and women at both time points (Table 2 and Figure 2Ai). Mean and median compliance were high in all age groups (Figure 2Ai). In total, 69% (43/62) and 56% (35/62) of the patients had missed only 0% to 5% of their expected injections at 6 and 12 months, respectively (Figure 2Bi). Adherence was markedly higher in men than in women (Table 2 and Figure 2Ci). At 6 months, adherence showed no clear difference across age groups, whereas at 12 months, it was lowest in the youngest age group (Figure 2Ci).

**Table 2:** Compliance, persistence, and adherence analyzed prospectively and retrospectively.

Characteristics				6 months							12 months				
				Total		Female		Male		Total			Female		Male
**Prospective analysis**															
	Patients, n		62		41		21		62			41		21	
	**Persistence, n (%)**														
		Yes	53 (85)		33 (80)		20 (95)		47 (76)			28 (68)		19 (90)	
		No	9 (15)		8 (20)		1 (5)		15 (24)			13 (32)		2 (10)	
	**Compliance (%)^a^**														
		Values, mean (SD)	93.6 (13.8)		91.2 (15.5)		97.7 (9)		92.6 (14.1)			90.6 (15.7)		95.4 (11.4)	
		Values, median (IQR)	100 (95.6-101.1)		97.8 (92.2-101.1)		101.1 (99.4-101.1)		98.9 (94.3-100)			97.3 (92.3-100)		99.7 (96.4-100)	
		Values, range	47.8-103.3		47.8-101.1		64.4-103.3		41.5-101.1			41.5-100.5		53-101.1	
	**Adherence, n (%)**														
		Yes	46 (74)		27 (66)		19 (90)		40 (65)			22 (54)		18 (86)	
		No	16 (26)		14 (34)		2 (10)		22 (35)			19 (46)		3 (14)	
**Retrospective analysis**															
	Patients, n		60		39		21		60			39		21	
	**Persistence, n (%)**														
		Yes	50 (83)		31 (79)		19 (90)		42 (70)			24 (62)		18 (86)	
		No	10 (17)		8 (21)		2 (10)		18 (30)			15 (38)		3 (14)	
	**Compliance (%)^a^**														
		Values, mean (SD)	96.7 (13)		95.7 (15.5)		98.4 (6.7)		96.8 (8.7)			95.8 (10.7)		98.2 (4.3)	
		Values, median (IQR)	101.1 (98.9-101.1)		101.1 (98.9-101.1)		100 (100-101.1)		100 (97.3-100)			100 (97.3-100)		99.7 (97.3-100)	
		Values, range	27.8-107.8		27.8-107.8		77.8-107.8		52.5-104.9			52.5-103.3		88-104.9	
	**Adherence, n (%)**														
		Yes	48 (80)		30 (77)		18 (86)		41 (68)			23 (59)		18 (86)	
		No	12 (20)		9 (23)		3 (14)		19 (32)			16 (41)		3 (14)	

^a^On the basis of the injection data up to 6 months and up to 12 months in patients with injection data available for the periods (prospective analysis: n=55 at 6 months and n=48 at 12 months; retrospective analysis: n=52 at 6 months and n=44 at 12 months).

In the retrospective analysis, persistence was 83% (50/60) at 6 months and markedly lower, 70% (42/60), at 12 months. It was higher in men than in women at 6 months (19/21, 90% vs 31/39, 79%) and 12 months (18/21, 86% vs 24/39, 62%) (Table 2). Mean and median compliance (based on injection data up to 6 months and up to 12 months in patients with injection data available for those time periods) were high in both men and women (Table 2 and Figure 2Aii) and in all age groups at both time points (Figure 2Aii). In total, 73% (44/60) and 62% (37/60) of the patients had missed only 0% to 5% of their expected injections at 6 and 12 months, respectively (Figure 2Bii). Adherence was higher in men than in women at both time points (Table 2 and Figure 2Cii). In the analysis by age group, those aged 50 to <60 years showed the lowest adherence at 6 months, whereas those aged <30 years showed the lowest adherence at 12 months (Figure 2Cii).

### HRQoL and Satisfaction With Treatment and Support

**Figure 3:**
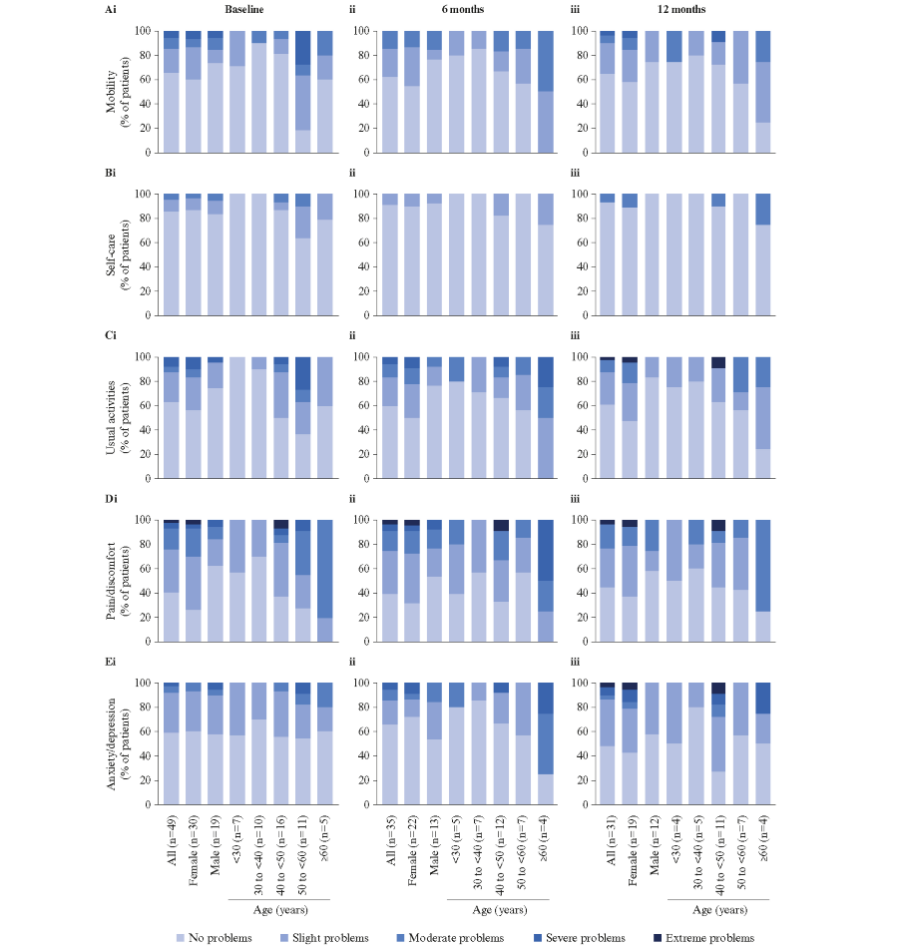
Patient-reported assessment of (A) mobility, (B) self-care, (C) usual activities, (D) pain/discomfort, and (E) anxiety/depression at (i) baseline, (ii) 6 months, and (iii) 12 months in the EuroQol 5-Dimension, 5-Level questionnaire (EQ-5D-5L).

In the EQ-5D-5L analysis, women reported more severe problems with mobility, usual activities, and pain/discomfort than men (Figure 3). None of the dimensions showed a consistent difference across age groups. The median (IQR) EQ-5D-5L index was 0.9 (0.8-1.0) at baseline and 0.9 (0.9-1.0) at 6 and 12 months. The median (IQR) visual analogue scale (VAS) was 81.0 (65.0-90.0) at baseline, 82.0 (62.0-90.0) at 6 months, and 80.0 (66.0-88.0) at 12 months. The EQ-5D-5L index and VAS showed no consistent differences between men and women or across age groups.

The median TSQM (version II) global satisfaction domain score remained stable during follow-up, showed no consistent difference between men and women, and was lowest in the youngest age group during follow-up (Table 3). Median domain scores for effectiveness and side effects showed no consistent differences between the age and gender subgroups. The median domain score for convenience was lower in women than in men and was lowest in the youngest age group (Table 3).

Most of the patients were satisfied or *neither satisfied nor dissatisfied* with the BETAPLUS PSDMP (Multimedia Appendix 2). Most patients were satisfied or *neither satisfied nor dissatisfied* with the BETACONNECT (Multimedia Appendix 3) and satisfied or very satisfied with the app (Multimedia Appendix 4). In analyses stratified by gender and age groups, the sample sizes in the respective strata were very small and did not allow for any further reliable conclusions (Multimedia Appendices 2-4).

The baseline EuroQol VAS score was higher in persistent and adherent patients than in nonpersistent and nonadherent patients (Multimedia Appendix 5). Logistic regression showed a significant association of the baseline EuroQol VAS score with persistence and adherence at 12 months but not at 6 months (Multimedia Appendix 6).

**Table 3:** Treatment Satisfaction Questionnaire for Medication (version II) domain scores.

Characteristics				Baseline				Month 6				Month 12		
				n (%)		Median (IQR)		n (%)		Median (IQR)		n (%)		Median (IQR)
**Effectiveness**														
	Total (N=62)			49 (79)		83.3 (66.7-91.7)		35 (56)		83.3 (58.3-91.7)		31 (50)		83.3 (58.3-100.0)
	**Gender**													
		Female (n=41)	30 (73)		83.3 (66.7-91.7)		22 (54)		83.3 (66.7-91.7)		19 (46)		83.3 (58.3-100.0)	
		Male (n=21)	19 (90)		75.0 (58.3-88.3)		13 (62)		83.3 (41.7-100.0)		12 (57)		83.3 (70.8-95.8)	
	**Age (years)**													
		<30 (n=9)	7 (78)		66.7 (66.7-100.0)		5 (56)		83.3 (75.0-91.7)		4 (44)		66.7 (41.7-83.3)	
		30 to <40 (n=15)	10 (67)		83.3 (83.3-83.3)		7 (47)		83.3 (75.0-91.7)		5 (33)		91.7 (83.3-91.7)	
		40 to <50 (n=19)	16 (84)		83.3 (79.2-91.7)		12 (63)		83.3 (50.0-91.7)		11 (58)		83.3 (66.7-100.0)	
		50 to <60 (n=13)	11 (85)		75.0 (50.0-91.7)		7 (54)		75.0 (25.0-100.0)		7 (54)		91.7 (25.0-100.0)	
		≥60 (n=6)	5 (83)		66.7 (58.3-66.7)		4 (67)		66.7 (54.2-79.2)		4 (67)		91.7 (70.8-100.0)	
**Side effects**														
	Total (N=62)			29 (47)		75.0 (58.3-83.3)		18 (29)		75.0 (58.3-87.5)		15 (24)		75.0 (75.0-91.7)
	**Gender**													
		Female (n=41)	16 (39)		70.8 (45.8-83.3)		10 (24)		75.0 (50.0-91.7)		7 (17)		75.0 (75.0-91.7)	
		Male (n=21)	13 (62)		75.0 (66.7-87.5)		8 (38)		75.0 (60.4-79.2)		8 (38)		75.0 (70.8-87.5)	
	**Age (years)**													
		<30 (n=9)	3 (33)		83.3 (41.7-83.3)		2 (22)		75.0 (75.0-75.0)		0 (0)		N/A^a^	
		30 to <40 (n=15)	7 (47)		75.0 (50.0-83.3)		4 (27)		54.2 (37.5-75.0)		2 (13)		75.0 (75.0-75.0)	
		40 to <50 (n=19)	8 (42)		70.8 (62.5-87.5)		6 (32)		66.7 (58.3-83.3)		5 (26)		83.3 (75.0-91.7)	
		50 to <60 (n=13)	7 (54)		83.3 (62.5-100.0)		4 (31)		81.3 (68.8-89.6)		5 (38)		75.0 (75.0-83.3)	
		≥60 (n=6)	4 (67)		62.5 (45.8-79.2)		2 (33)		87.5 (75.0-100.0)		3 (50)		75.0 (58.3-91.7)	
**Convenience**														
	Total (N=62)			49 (79)		72.2 (61.1-83.3)		35 (56)		77.8 (66.7-88.9)		31 (50)		72.2 (61.1-88.9)
	**Gender**													
		Female (n=41)	30 (73)		69.4 (61.1-77.8)		22 (54)		75.0 (55.6-83.3)		19 (46)		72.2 (50.0-88.9)	
		Male (n=21)	19 (90)		77.8 (66.7-88.9)		13 (62)		83.3 (77.8-88.9)		12 (57)		77.8 (66.7-86.1)	
	**Age (years)**													
		<30 (n=9)	7 (78)		66.7 (55.6-72.2)		5 (56)		55.6 (22.2-66.7)		4 (44)		47.2 (36.1-55.6)	
		30 to <40 (n=15)	10 (67)		66.7 (61.1-77.8)		7 (47)		77.8 (66.7-77.8)		5 (33)		72.2 (66.7-83.3)	
		40 to <50 (n=19)	16 (84)		72.2 (66.7-83.3)		12 (63)		83.3 (69.4-88.9)		11 (58)		77.8 (50.0-88.9)	
		50 to <60 (n=13)	11 (85)		88.9 (72.2-94.4)		7 (54)		88.9 (77.8-94.4)		7 (54)		88.9 (77.8-94.4)	
		≥60 (n=6)	5 (83)		72.2 (61.1-72.2)		4 (67)		75.0 (58.3-91.7)		4 (67)		72.2 (63.9-80.6)	
**Global satisfaction**														
	Total (N=62)			49 (79)		83.3 (66.7-91.7)		35 (56)		83.3 (66.7-91.7)		31 (50)		83.3 (66.7-91.7)
	**Gender**													
		Female (n=41)	30 (73)		83.3 (66.7-91.7)		22 (54)		83.3 (66.7-91.7)		19 (46)		75.0 (58.3-91.7)	
		Male (n=21)	19 (90)		83.3 (66.7-91.7)		13 (62)		75.0 (66.7-91.7)		12 (57)		83.3 (70.8-91.7)	
	**Age (years)**													
		<30 (n=9)	7 (78)		75.0 (66.7-83.3)		5 (56)		66.7 (58.3-91.7)		4 (44)		58.3 (54.2-66.7)	
		30 to <40 (n=15)	10 (67)		83.3 (66.7-83.3)		7 (47)		75.0 (66.7-83.3)		5 (33)		83.3 (75.0-91.7)	
		40 to <50 (n=19)	16 (84)		79.2 (66.7-87.5)		12 (63)		79.2 (66.7-91.7)		11 (58)		83.3 (66.7-83.3)	
		50 to <60 (n=13)	11 (85)		83.3 (66.7-100.0)		7 (54)		83.3 (66.7-100.0)		7 (54)		91.7 (83.3-100.0)	
		≥60 (n=6)	5 (83)		75.0 (66.7-75.0)		4 (67)		79.2 (66.7-91.7)		4 (67)		79.2 (70.8-83.3)	

^a^N/A: not applicable.

Baseline TSQM (version II) domain scores for effectiveness, side effects, convenience, and global satisfaction were higher in persistent and adherent patients than in nonpersistent and nonadherent patients (Multimedia Appendix 7). Logistic regression showed a significant association between the baseline side effects score and persistence at 12 months, between the baseline convenience score and adherence at 6 and 12 months, and between the baseline global satisfaction score and adherence at 6 and 12 months (Multimedia Appendix 6).

The proportion of patients who were satisfied or very satisfied with the PSDMP at baseline was higher in persistent patients than in nonpersistent patients, whereas it was similar in adherent and nonadherent patients (Multimedia Appendix 8). Regarding the BETACONNECT and the app, the proportion of patients who were satisfied or very satisfied at baseline was higher in persistent and adherent patients than in nonpersistent and nonadherent patients (Multimedia Appendix 8). Logistic regression analyses did not suggest significant associations of persistence and adherence with satisfaction with the PSDMP, BETACONNECT, or app (Multimedia Appendix 6).

### PEAK Use

The proportion of patients using PEAK at least once was higher among men (14/21, 67%) than among women (20/41, 49%) and was lowest among those aged ≥60 years (1/6, 17% compared with 6/9, 67%; 9/15, 60%; 10/19, 53%; and 8/13, 62% among those aged <30; 30 to <40; 40 to <50; and 50 to <60 years, respectively).

In an analysis of PEAK use over 3-month intervals, the number of active days (days in which PEAK was used at least once) and the average number of games played per week varied widely between patients in each interval (Table 4).

The 10 most popular games included four games in the language category (Word Hunt, Babble Bots, Word Fresh, and Grow), two in the focus category (Must Sort and Objectifind), two in the problem-solving category (Pixel Logic and Low Pop), one in the memory category (Perilous Path), and one in the mental agility category (Turtle Traffic). Of the 10 most popular games, Pixel Logic was played for the longest cumulative time during each 3-month interval (median 37, IQR 3-107 minutes at months 1-3; median 6, IQR 0-99 minutes at months 4-6; median 11, IQR 0-55 minutes at months 7-9; and median 25, IQR 2-59 minutes at months 10-12).

**Table 4:** PEAK use over 3-month intervals.

Characteristics			Values (N=62)				
			n (%)		Median (IQR)		Range
**Number of days active per patient^a^**							
	Data not assigned to any time interval	12 (19)		5 (2-37)		1-83	
	Months 1-3	32 (52)		23 (8-49)		1-91	
	Months 4-6	18 (29)		4 (1-68)		1-91	
	Months 7-9	14 (23)		14 (1-60)		1-81	
	Months 10-12	14 (23)		10 (2-27)		2-89	
**Number of games played per patient per week^b^**							
	Data not assigned to any time interval	12 (19)		0 (0-8)		0-33	
	Months 1-3	32 (52)		5 (1-12)		0-158	
	Months 4-6	18 (29)		0 (0-11)		0-218	
	Months 7-9	14 (23)		2 (0-10)		0-259	
	Months 10-12	14 (23)		4 (0-7)		0-116	

^a^An active day was defined as a day on which PEAK was used at least once.

^b^Only the 10 most frequently played games (all PEAK users) were counted. A single game could be played multiple times per day.

## Discussion

### Principal Findings

This study builds on the results of a previous pilot study [5], performing follow-up for 1 year, and incorporating completion of PRO instruments.

The proportion of patients participating was 4.44% (79/1778) of registered accounts for the app. More than three-quarters (48/62, 77%) of the participants had ≥12 months of injection data for prospective analysis. Compliance over the 1-year prospective observational period was high (median 98.9%, IQR 94.3%-100%), with similar percentages among men and women. Persistence and adherence decreased from 85% (53/62) and 74% (46/62), respectively, at 6 months to 76% (47/62) and 65% (40/62), respectively, at 12 months, and were higher in men than in women in the prospective analysis.

The PRO questionnaires were answered by 79% (49/62) of the participants at baseline and 50% (31/62) of the participants at month 12. An immediate reward for participation rather than compensation at the end of the study may have increased the completion rate of the PRO questionnaires [11,12]. Women reported more severe problems than men in some EQ-5D-5L dimensions (mobility, usual activities, and pain/discomfort) and had lower median domain scores than men for convenience in the TSQM (version II). HRQoL showed no consistent changes across age groups. Convenience and global satisfaction (assessed using the TSQM [version II]) were lowest in the youngest age group. Persistence and adherence at 12 months were associated with baseline HRQoL (EuroQol VAS) and satisfaction with treatment (scores in specific domains of the TSQM [version II]) but not satisfaction with support.

PEAK was used at least once by 67% (14/21) of the male participants and by 49% (20/41) of the female participants. The frequency of PEAK use varied widely among patients.

### Comparison With Previous Work

This study used the same digital approach as the previous pilot study [5] but recorded data over a longer period (1 year prospectively and ≤1 year retrospectively compared with 6 months in total in the pilot study) and allowed comparison of medication-taking behavior between retrospective and prospective study periods. In addition, this study recorded PROs and tested the acceptance of an optional cognitive training tool (ie, it involved active engagement by the participants), whereas the pilot study used only basic registration data and routinely recorded injection data.

The pattern of decreasing persistence and adherence with continuing high compliance over the 1-year follow-up period appeared in both the prospective and retrospective analyses. These findings confirm previous reports that an increasing number of patients stop their treatment over time [13-16], with substantial attrition occurring early in the treatment course, within 1 year [15,16]. In contrast, the high compliance at both 6 and 12 months suggests that patients remaining on treatment take their medication very reliably. The similarity in persistence, compliance, and adherence between the prospective and retrospective analyses argues against an observer effect prompting patients to act differently once they were aware that they were participating in a study (ie, after providing consent).

Persistence and adherence in the prospective observational period (53/62, 85% and 46/62, 74%, respectively, at 6 months) were lower than those reported in the pilot study (90/94, 96% and 84/94, 89%, respectively) [5]. This may be explained by patient motivation and stamina, which may be higher in a 6‑month study than in a 1-year study.

Men appeared more persistent and more adherent than women in both prospective and retrospective analyses. We cannot say whether this is a valid finding or a result of confounding; for example, by men being more technophile than women. In this context, more women may have chosen to stop documenting injections in the app over time, which by definition would have resulted in their classification as nonpersistent and nonadherent thereafter.

Persistence and adherence showed little difference between men and women in the pilot study [5] and in an observational study of patients receiving interferon *β*-1b via the BETACONNECT autoinjector in Germany (the BETAPREDICT study) [13]. However, several large studies of patients with MS have shown greater adherence in men than in women [17-19], consistent with our current findings. A US-based administrative claims database study of 648 patients with MS showed that those who were adherent to disease-modifying therapy over a study period of ≥24 months were more likely to be men than in nonadherent patients (173/448, 38.6% vs 52/200, 26%; *P*=.002) [17]. Another US-based study used enrollment and claims data from an upper Midwest health plan between 2011 and 2013 and showed that female patients were 5.5% less likely to be adherent than male patients [18]. A third US-based claims database study of 8382 patients with MS followed for 12 months found that male patients had an increased likelihood of adherence to disease-modifying therapy (odds ratio 1.2, 95% CI 1.085-1.335; *P*<.001) [19].

We found the lowest rates of adherence and global treatment satisfaction in the youngest age group (<30 years) in the prospective analysis. In the pilot study, those aged 30 to 39 years had the lowest rate of adherence [5]. Although age thresholds vary across studies, these results are in general agreement with other studies reporting higher rates of adherence in older patients [17-19]. A US-based administrative claims database study showed that patients with MS who were adherent to disease-modifying therapy were on average older than those who were nonadherent (mean 43.5, SD 8.0 years vs mean 41.8, SD 8.1 years; *P*=.02) [17]. A US-based study using health plan data showed that patients aged >45 years were 13.7% to 18.6% more likely to be adherent than younger patients [18]. A third US-based claims database study found that age groups older than 18-34 years had an increased likelihood of adherence to disease-modifying therapy (odds ratios 1.220-1.331; *P*<.001 to *P*=.001) [19].

Women reported problems with mobility, usual activities, and pain/discomfort more frequently than men in our analysis. A previous study of 144 patients (99 women and 45 men) with MS in Germany also found that women reported problems with usual activities more frequently than men, although the association was not significant in multivariate analysis [20]. In contrast, a large UK-based study of 4516 patients with MS (including 3198 women and 1301 men) showed that men reported problems with mobility, self-care, and usual activities more frequently than women [21]. The reasons for the inconsistency between studies are unclear but could include differences in study design and location.

There was a steep attrition of participants’ use of the PEAK app after 3 months, as also seen for other apps [22]. Free access to the professional version of the brain training tool did not provide enough incentive for participants to continue using it over the entire study period. Hence, voluntary and free access to PEAK does not appear to be an effective approach to provide constant brain training over a longer period. Peer-to-peer interactions (eg, via social media challenges, multiplayer games, or guided training programs) may have a positive impact on long-term engagement with gamified apps [23-25].

### Limitations

The generalizability of the results is limited by the small sample size and the possibility of selection bias. The number of patients was lower than in the pilot study [5], and it is possible that mainly technophile patients agreed to participate. We were unable to compare app users who participated in the study with those who did not, because demographic and clinical data from the latter group were not available.

Although we provided incentives (vouchers) for the completion of each questionnaire after the end of the observation period, the number of participants still decreased over the 1-year period. More immediate ways to incentivize patients directly after completing the questionnaires may be warranted to encourage continued active study participation.

Only patients using interferon *β*-1b were eligible to participate in this study. However, the aim of our study was to describe medication-taking behavior and collect PROs among patients treated with interferon *β*-1b and not to compare their behavior with that of other patient groups. In the future, open platforms that do not restrict participation to users of certain drugs will be more versatile in terms of patient pool, study design, and indication than drug-specific platforms. IDS and Bayer have codeveloped a data capture tool, the my ePRO app, which can be used for stand-alone studies or in association with randomized controlled trials and observational studies. The usability of the my ePRO app was assessed in the DePRO study [26]. The DePRO study showed that digitally authenticating eligible patients, with participants registering by scanning the 2D matrix code on the outer packaging of their prescribed medication (a standard feature of prescription medications in the European Union) is a feasible approach for a digital study (manuscript in preparation).

We were unable to perform source data verification or clarify incomplete data from individual patients because the analysis results were anonymized. We were also unable to assess whether offering access to PEAK had any influence on adherence or commitment to the study because of the lack of a control group. Other limitations are similar to those described for the pilot study [5], including the use of nonvalidated technology to obtain data on medication-taking behavior, missing data (which have not been replaced), and the inability to distinguish between patients discontinuing treatment and those simply ceasing to document their injections.

### Conclusions

This study showed high compliance and decreasing persistence and adherence over 1 year and demonstrated the feasibility of including remotely completed electronic PRO instruments in digital observational studies. The feasibility and influence of gamification in this setting remain unclear.

## Appendix

Multimedia Appendix 1 Service questionnaire.

Multimedia Appendix 2 Responses to service questionnaire part 1: “Are you satisfied with the BETAPLUS patient support program?”.

Multimedia Appendix 3 Responses to service questionnaire part 2: “Are you satisfied with the BETACONNECT autoinjector?”.

Multimedia Appendix 4 Responses to service questionnaire part 3: “Are you satisfied with the myBETAapp?”.

Multimedia Appendix 5 Baseline EuroQol 5-Dimension, 5-Level questionnaire (EQ-5D-5L) visual analogue scale stratified by persistence and adherence at 6 and 12 months.

Multimedia Appendix 6 Association of persistence and adherence at 6 and 12 months with baseline health-related quality of life and satisfaction with treatment and support.

Multimedia Appendix 7 Baseline Treatment Satisfaction Questionnaire for Medication (version II) domain scores stratified by persistence and adherence at 6 and 12 months.

Multimedia Appendix 8 Baseline satisfaction with the Patient Support and Disease Management Program, BETACONNECT, and app stratified by persistence and adherence at 6 and 12 months.

## Abbreviations

HRQoL: health-related quality of life
IDS: Institut Dr. Schauerte
MS: multiple sclerosis
PRO: patient-reported outcome
PSDMP: Patient Support and Disease Management Program
TSQM: Treatment Satisfaction Questionnaire for Medication
VAS: visual analogue scale
