# Correction: Ascertaining Medication Use and Patient-Reported Outcomes via an App and Exploring Gamification in Patients With Multiple Sclerosis Treated With Interferon β-1b: Observational Study

**DOI:** 10.2196/38002

**Published:** 2022-03-17

**Authors:** Volker Limmroth, Kirsten Bayer-Gersmann, Christian Mueller, Markus Schürks

**Affiliations:** 1 Clinic for Neurology and Palliative Medicine Municipal Hospital Köln-Merheim Cologne Germany; 2 Institut Dr. Schauerte Munich Germany; 3 Bayer Vital GmbH Leverkusen Germany

In “Ascertaining Medication Use and Patient-Reported Outcomes via an App and Exploring Gamification in Patients With Multiple Sclerosis Treated With Interferon β-1b: Observational Study” (JMIR Form Res 2022;6(3):e31972) the authors noted one error.

In the originally published article, Figure 2 appeared incorrectly (as shown in the Multimedia Appendix 1).

The correct Figure 2 is provided below.

The correction will appear in the online version of the paper on the JMIR Publications website on March 17, 2022, together with the publication of this correction notice. Because this was made after submission to PubMed, PubMed Central, and other full-text repositories, the corrected article has also been resubmitted to those repositories.

**Figure 2:**
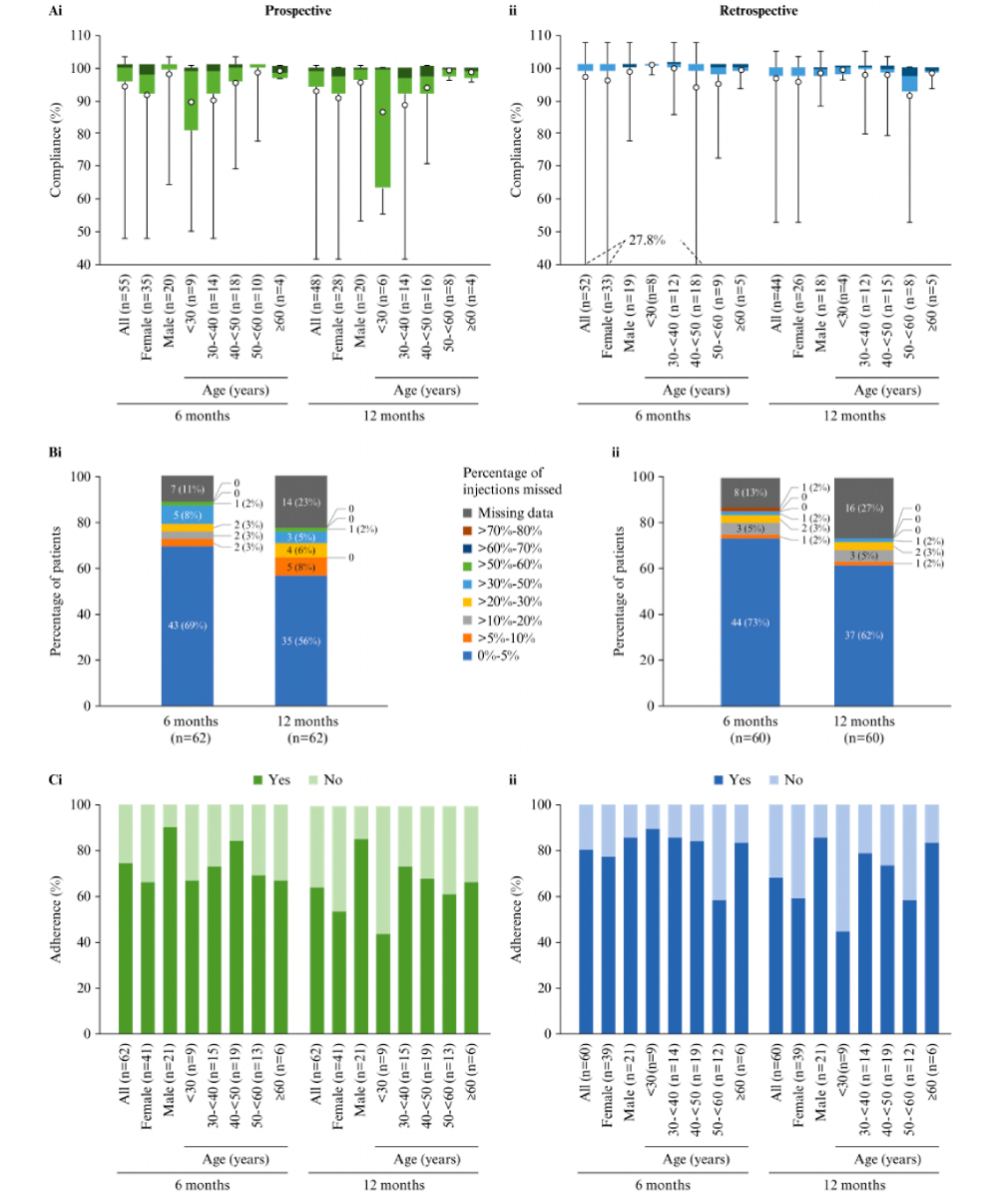
(A) Compliance overall and by gender and age group, (B) patients categorized by percentage of interferon β-1b injections missed (based on an expected frequency of 1 injection every other day), and (C) adherence overall and by gender and age group, analyzed (i) prospectively and (ii) retrospectively. Compliance at 6 and 12 months was assessed in patients with ≥6 and ≥12 months of injection-related data, respectively. In the box and whisker plots, the colored bars indicate median and IQR, the whiskers indicate minimum and maximum values, and the white circles indicate the mean.

## Appendix

Multimedia Appendix 1 Originally published Figure 2.

