# Supplementary material for: Ascertaining Medication Use and Patient-Reported Outcomes via an App and Exploring Gamification in Patients With Multiple Sclerosis Treated With Interferon β-1b: Observational Study
Source: JMIR Form Res. 2022 Mar 14;6(3):e31972. doi: 10.2196/31972 (PMC8929528; doi:10.2196/31972)
Supplement: Multimedia Appendix 1 [file formative_v6i3e31972_app1.doc]

## Multimedia Appendix

# Ascertaining Medication Use and Patient-Reported Outcomes Via an App and Exploring Gamification in Patients With Multiple Sclerosis Treated With Interferon *β*-1b: Observational Study

Volker Limmroth, MD; Kirsten Bayer-Gersmann, BEng; Christian Müller, PhD; Markus Schürks, MD, MSc

## Service Questionnaire

1. Are you satisfied with the BETAPLUS patient support program?

*This question can be answered on a 5-point Likert Scale (very satisfied/satisfied/neither satisfied nor dissatisfied/dissatisfied/very dissatisfied) also including the option “not participating”.*

2. Are you satisfied with the BETACONNECT?

*This question can be answered on a 5-point Likert Scale (very satisfied/satisfied/neither satisfied nor dissatisfied/dissatisfied/very dissatisfied) also including the option “not using”.*

3. Are you satisfied with myBETAapp?

*This question can be answered on a 5-point Likert Scale (very satisfied/satisfied/neither satisfied nor dissatisfied/dissatisfied/very dissatisfied).*
