# Supplementary material for: Ascertaining Medication Use and Patient-Reported Outcomes via an App and Exploring Gamification in Patients With Multiple Sclerosis Treated With Interferon β-1b: Observational Study
Source: JMIR Form Res. 2022 Mar 14;6(3):e31972. doi: 10.2196/31972 (PMC8929528; doi:10.2196/31972)
Supplement: Multimedia Appendix 4 [file formative_v6i3e31972_app4.doc]

## Multimedia Appendix

# Ascertaining Medication Use and Patient-Reported Outcomes Via an App and Exploring Gamification in Patients With Multiple Sclerosis Treated With Interferon *β*-1b: Observational Study

Volker Limmroth, MD; Kirsten Bayer-Gersmann, BEng; Christian Müller, PhD; Markus Schürks, MD, MSc

**Table.** Responses to service questionnaire part 3: “Are you satisfied with the myBETAapp?”

|  | | **Baseline (n=49)** | | **Month 3 (n=36)** | | **Month 6 (n=35)** | | **Month 9 (n=32)** | | **Month 12 (n=31)** | |
| --- | --- | --- | --- | --- | --- | --- | --- | --- | --- | --- | --- |
|  | | **n** | **(%)** | **n** | **(%)** | **n** | **(%)** | **n** | **(%)** | **n** | **(%)** |
| **Total** | | 49 | (100) | 36 | (100) | 35 | (100) | 32 | (100) | 31 | (100) |
|  | Very satisfied | 16 | (33) | 12 | (33) | 13 | (37) | 13 | (41) | 14 | (45) |
|  | Satisfied | 23 | (47) | 17 | (47) | 17 | (49) | 14 | (44) | 12 | (39) |
|  | Neither satisfied nor dissatisfied | 6 | (12) | 3 | (8) | 3 | (9) | 2 | (6) | 2 | (6) |
|  | Dissatisfied | 3 | (6) | 2 | (6) | 0 | – | 1 | (3) | 1 | (3) |
|  | Very dissatisfied | 1 | (2) | 2 | (6) | 2 | (6) | 2 | (6) | 2 | (6) |
| **Female** | | 30 | (100) | 22 | (100) | 22 | (100) | 19 | (100) | 19 | (100) |
|  | Very satisfied | 9 | (30) | 8 | (36) | 8 | (36) | 8 | (42) | 8 | (42) |
|  | Satisfied | 15 | (50) | 10 | (45) | 10 | (46) | 7 | (37) | 7 | (37) |
|  | Neither satisfied nor dissatisfied | 4 | (13) | 2 | (9) | 2 | (9) | 1 | (5) | 1 | (5) |
|  | Dissatisfied | 1 | (3) | 0 | – | 0 | – | 1 | (5) | 1 | (5) |
|  | Very dissatisfied | 1 | (3) | 2 | (9) | 2 | (9) | 2 | (11) | 2 | (11) |
| **Male** | | 19 | (100) | 14 | (100) | 13 | (100) | 13 | (100) | 12 | (100) |
|  | Very satisfied | 7 | (37) | 4 | (29) | 5 | (38) | 5 | (38) | 6 | (50) |
|  | Satisfied | 8 | (42) | 7 | (50) | 7 | (54) | 7 | (54) | 5 | (42) |
|  | Neither satisfied nor dissatisfied | 2 | (11) | 1 | (7) | 1 | (8) | 1 | (8) | 1 | (8) |
|  | Dissatisfied | 2 | (11) | 2 | (14) | 0 | – | 0 | – | 0 | – |
|  | Very dissatisfied | 0 | – | 0 | – | 0 | – | 0 | – | 0 | – |
| **<30 years** | | 7 | (100) | 4 | (100) | 5 | (100) | 4 | (100) | 4 | (100) |
|  | Very satisfied | 0 | – | 0 | – | 1 | (20) | 1 | (25) | 1 | (25) |
|  | Satisfied | 4 | (57) | 2 | (50) | 3 | (60) | 1 | (25) | 2 | (50) |
|  | Neither satisfied nor dissatisfied | 1 | (14) | 1 | (25) | 0 | – | 0 | – | 0 | – |
|  | Dissatisfied | 2 | (29) | 0 | – | 0 | – | 1 | (25) | 0 | – |
|  | Very dissatisfied | 0 | – | 1 | (25) | 1 | (20) | 1 | (25) | 1 | (25) |
| **30 to <40 years** | | 10 | (100) | 8 | (100) | 7 | (100) | 6 | (100) | 5 | (100) |
|  | Very satisfied | 3 | (30) | 1 | (13) | 2 | (29) | 0 | – | 1 | (20) |
|  | Satisfied | 6 | (60) | 6 | (75) | 5 | (71) | 6 | (100) | 3 | (60) |
|  | Neither satisfied nor dissatisfied | 0 | – | 0 | – | 0 | – | 0 | – | 1 | (20) |
|  | Dissatisfied | 1 | (10) | 1 | (13) | 0 | – | 0 | – | 0 | – |
|  | Very dissatisfied | 0 | – | 0 | – | 0 | – | 0 | – | 0 | – |
| **40 to <50 years** | | 16 | (100) | 12 | (100) | 12 | (100) | 12 | (100) | 11 | (100) |
|  | Very satisfied | 3 | (19) | 4 | (33) | 2 | (17) | 4 | (33) | 6 | (55) |
|  | Satisfied | 9 | (56) | 5 | (42) | 6 | (50) | 5 | (42) | 3 | (27) |
|  | Neither satisfied nor dissatisfied | 3 | (19) | 1 | (8) | 3 | (25) | 2 | (17) | 1 | (9) |
|  | Dissatisfied | 0 | – | 1 | (8) | 0 | – | 0 | – | 0 | – |
|  | Very dissatisfied | 1 | (6) | 1 | (8) | 1 | (8) | 1 | (8) | 1 | (9) |
| **50 to <60 years** | | 11 | (100) | 7 | (100) | 7 | (100) | 6 | (100) | 7 | (100) |
|  | Very satisfied | 8 | (73) | 5 | (71) | 6 | (86) | 6 | (100) | 5 | (71) |
|  | Satisfied | 2 | (18) | 1 | (14) | 1 | (14) | 0 | – | 2 | (29) |
|  | Neither satisfied nor dissatisfied | 1 | (9) | 1 | (14) | 0 | – | 0 | – | 0 | – |
|  | Dissatisfied | 0 | – | 0 | – | 0 | – | 0 | – | 0 | – |
|  | Very dissatisfied | 0 | – | 0 | – | 0 | – | 0 | – | 0 | – |
| **≥60 years** | | 5 | (100) | 5 | (100) | 4 | (100) | 4 | (100) | 4 | (100) |
|  | Very satisfied | 2 | (40) | 2 | (40) | 2 | (50) | 2 | (50) | 1 | (25) |
|  | Satisfied | 2 | (40) | 3 | (60) | 2 | (50) | 2 | (50) | 2 | (50) |
|  | Neither satisfied nor dissatisfied | 1 | (20) | 0 | – | 0 | – | 0 | – | 0 | – |
|  | Dissatisfied | 0 | – | 0 | – | 0 | – | 0 | – | 1 | (25) |
|  | Very dissatisfied | 0 | – | 0 | – | 0 | – | 0 | – | 0 | –49 |
