# Supplementary material for: Ascertaining Medication Use and Patient-Reported Outcomes via an App and Exploring Gamification in Patients With Multiple Sclerosis Treated With Interferon β-1b: Observational Study
Source: JMIR Form Res. 2022 Mar 14;6(3):e31972. doi: 10.2196/31972 (PMC8929528; doi:10.2196/31972)
Supplement: Multimedia Appendix 5 [file formative_v6i3e31972_app5.doc]

## Multimedia Appendix

# Ascertaining Medication Use and Patient-Reported Outcomes Via an App and Exploring Gamification in Patients With Multiple Sclerosis Treated With Interferon *β*-1b: Observational Study

Volker Limmroth, MD; Kirsten Bayer-Gersmann, BEng; Christian Müller, PhD; Markus Schürks, MD, MSc

**Table.** Baseline EQ-5D-5L VAS stratified by persistence and adherence at 6 months and 12 months

|  |  |  |  | **Baseline EQ-5D-5L VAS** | | | | | | | |
| --- | --- | --- | --- | --- | --- | --- | --- | --- | --- | --- | --- |
|  |  |  |  | **Non-missing** | **Mean** | **SD** | **Min** | **Q1** | **Median** | **Q3** | **Max** |
|  | Persistence | | |  |  |  |  |  |  |  |  |
|  |  | 6 months | |  |  |  |  |  |  |  |  |
|  |  |  | Total | 49 | 74.9 | 19.6 | 21.0 | 65.0 | 81.0 | 90.0 | 99.0 |
|  |  |  | Yes | 44 | 76.5 | 18.2 | 27.0 | 68.5 | 83.5 | 90.0 | 99.0 |
|  |  |  | No | 5 | 61.0 | 28.2 | 21.0 | 44.0 | 70.0 | 80.0 | 90.0 |
|  |  | 12 months | |  |  |  |  |  |  |  |  |
|  |  |  | Total | 49 | 74.9 | 19.6 | 21.0 | 65.0 | 81.0 | 90.0 | 99.0 |
|  |  |  | Yes | 40 | 80.2 | 14.7 | 40.0 | 75.0 | 85.0 | 90.0 | 99.0 |
|  |  |  | No | 9 | 51.6 | 22.4 | 21.0 | 42.0 | 45.0 | 70.0 | 85.0 |
|  | Adherence | | |  |  |  |  |  |  |  |  |
|  |  | 6 months | |  |  |  |  |  |  |  |  |
|  |  |  | Total | 49 | 74.9 | 19.6 | 21.0 | 65.0 | 81.0 | 90.0 | 99.0 |
|  |  |  | Yes | 39 | 77.6 | 17.7 | 27.0 | 75.0 | 85.0 | 90.0 | 99.0 |
|  |  |  | No | 10 | 64.7 | 24.2 | 21.0 | 45.0 | 66.5 | 89.0 | 94.0 |
|  |  | 12 months | |  |  |  |  |  |  |  |  |
|  |  |  | Total | 49 | 74.9 | 19.6 | 21.0 | 65.0 | 81.0 | 90.0 | 99.0 |
|  |  |  | Yes | 35 | 80.6 | 14.2 | 40.0 | 75.0 | 85.0 | 90.0 | 99.0 |
|  |  |  | No | 14 | 60.8 | 24.2 | 21.0 | 44.0 | 57.0 | 85.0 | 94.0 |

EQ-5D-5L VAS: visual analogue scale of the EuroQol 5-Dimension, 5-Level questionnaire; max: maximum; min: minimum; Q: quartile.
