# Supplementary material for: Ascertaining Medication Use and Patient-Reported Outcomes via an App and Exploring Gamification in Patients With Multiple Sclerosis Treated With Interferon β-1b: Observational Study
Source: JMIR Form Res. 2022 Mar 14;6(3):e31972. doi: 10.2196/31972 (PMC8929528; doi:10.2196/31972)
Supplement: Multimedia Appendix 6 [file formative_v6i3e31972_app6.doc]

## Multimedia Appendix

# Ascertaining Medication Use and Patient-Reported Outcomes Via an App and Exploring Gamification in Patients With Multiple Sclerosis Treated With Interferon *β*-1b: Observational Study

Volker Limmroth, MD; Kirsten Bayer-Gersmann, BEng; Christian Müller, PhD; Markus Schürks, MD, MSc


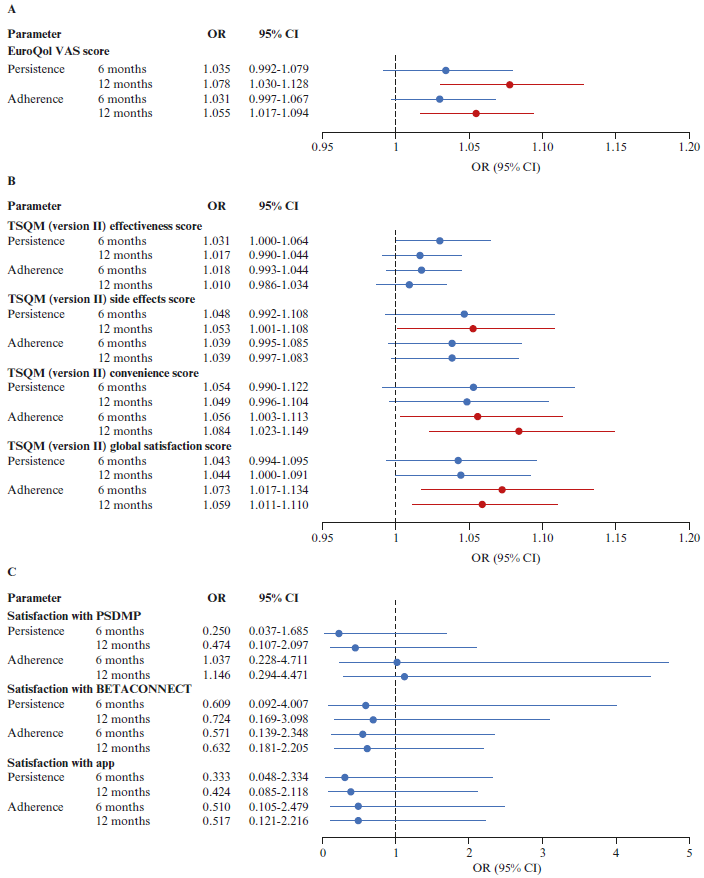


**Figure.** Association of persistence and adherence at 6 months and 12 months with baseline health-related quality of life and satisfaction with treatment and support. Associations with (A) EuroQol VAS score, (B) TSQM (version II) domain scores, and (C) satisfaction with support at baseline (‘neither satisfied nor dissatisfied’ or dissatisfied or very dissatisfied versus satisfied or very satisfied) are shown. Red data points indicate statistically significant ORs. OR: odds ratio; PSDMP: Patient Support and Disease Management Program; TSQM: Treatment Satisfaction Questionnaire for Medication; VAS: visual analogue scale.
