# Supplementary material for: Ascertaining Medication Use and Patient-Reported Outcomes via an App and Exploring Gamification in Patients With Multiple Sclerosis Treated With Interferon β-1b: Observational Study
Source: JMIR Form Res. 2022 Mar 14;6(3):e31972. doi: 10.2196/31972 (PMC8929528; doi:10.2196/31972)
Supplement: Multimedia Appendix 7 [file formative_v6i3e31972_app7.doc]

## Multimedia Appendix

# Ascertaining Medication Use and Patient-Reported Outcomes Via an App and Exploring Gamification in Patients With Multiple Sclerosis Treated With Interferon *β*-1b: Observational Study

Volker Limmroth, MD; Kirsten Bayer-Gersmann, BEng; Christian Müller, PhD; Markus Schürks, MD, MSc

**Table.** Baseline TSQM (version II) domain scores stratified by persistence and adherence at 6 months and 12 months

|  |  |  |  | **Baseline TSQM (version II) domain score** | | | | | | | |
| --- | --- | --- | --- | --- | --- | --- | --- | --- | --- | --- | --- |
|  |  |  |  | **Non-missing** | **Mean** | **SD** | **Min** | **Q1** | **Median** | **Q3** | **Max** |
| **Effectiveness** | | | | | | | | | | | |
|  | Persistence | | |  |  |  |  |  |  |  |  |
|  |  | 6 months | |  |  |  |  |  |  |  |  |
|  |  |  | Total | 49 | 72.6 | 25.3 | 0.0 | 66.7 | 83.3 | 91.7 | 100.0 |
|  |  |  | Yes | 44 | 75.2 | 24.7 | 0.0 | 66.7 | 83.3 | 91.7 | 100.0 |
|  |  |  | No | 5 | 50.0 | 20.4 | 16.7 | 50.0 | 50.0 | 66.7 | 66.7 |
|  |  | 12 months | |  |  |  |  |  |  |  |  |
|  |  |  | Total | 49 | 72.6 | 25.3 | 0.0 | 66.7 | 83.3 | 91.7 | 100.0 |
|  |  |  | Yes | 40 | 74.8 | 25.4 | 0.0 | 66.7 | 83.3 | 91.7 | 100.0 |
|  |  |  | No | 9 | 63.0 | 23.6 | 16.7 | 50.0 | 66.7 | 75.0 | 100.0 |
|  | Adherence | | |  |  |  |  |  |  |  |  |
|  |  | 6 months | |  |  |  |  |  |  |  |  |
|  |  |  | Total | 49 | 72.6 | 25.3 | 0.0 | 66.7 | 83.3 | 91.7 | 100.0 |
|  |  |  | Yes | 39 | 75.2 | 25.4 | 0.0 | 66.7 | 83.3 | 91.7 | 100.0 |
|  |  |  | No | 10 | 62.5 | 23.3 | 16.7 | 50.0 | 62.5 | 83.3 | 100.0 |
|  |  | 12 months | |  |  |  |  |  |  |  |  |
|  |  |  | Total | 49 | 72.6 | 25.3 | 0.0 | 66.7 | 83.3 | 91.7 | 100.0 |
|  |  |  | Yes | 35 | 74.5 | 26.4 | 0.0 | 66.7 | 83.3 | 91.7 | 100.0 |
|  |  |  | No | 14 | 67.9 | 22.4 | 16.7 | 50.0 | 66.7 | 83.3 | 100.0 |
| **Side effects^a^** | | | | | | | | | | | |
|  | Persistence | | |  |  |  |  |  |  |  |  |
|  |  | 6 months | |  |  |  |  |  |  |  |  |
|  |  |  | Total | 29 | 70.1 | 22.6 | 8.3 | 58.3 | 75.0 | 83.3 | 100.0 |
|  |  |  | Yes | 26 | 72.8 | 19.7 | 33.3 | 58.3 | 75.0 | 83.3 | 100.0 |
|  |  |  | No | 3 | 47.2 | 37.6 | 8.3 | 8.3 | 50.0 | 83.3 | 83.3 |
|  |  | 12 months | |  |  |  |  |  |  |  |  |
|  |  |  | Total | 29 | 70.1 | 22.6 | 8.3 | 58.3 | 75.0 | 83.3 | 100.0 |
|  |  |  | Yes | 24 | 74.5 | 19.3 | 33.3 | 62.5 | 75.0 | 85.4 | 100.0 |
|  |  |  | No | 5 | 49.2 | 27.7 | 8.3 | 41.7 | 50.0 | 62.5 | 83.3 |
|  | Adherence | | |  |  |  |  |  |  |  |  |
|  |  | 6 months | |  |  |  |  |  |  |  |  |
|  |  |  | Total | 29 | 70.1 | 22.6 | 8.3 | 58.3 | 75.0 | 83.3 | 100.0 |
|  |  |  | Yes | 23 | 74.1 | 20.6 | 33.3 | 58.3 | 75.0 | 87.5 | 100.0 |
|  |  |  | No | 6 | 54.9 | 25.3 | 8.3 | 50.0 | 60.4 | 66.7 | 83.3 |
|  |  | 12 months | |  |  |  |  |  |  |  |  |
|  |  |  | Total | 29 | 70.1 | 22.6 | 8.3 | 58.3 | 75.0 | 83.3 | 100.0 |
|  |  |  | Yes | 21 | 75.2 | 20.2 | 33.3 | 66.7 | 75.0 | 87.5 | 100.0 |
|  |  |  | No | 8 | 56.8 | 24.4 | 8.3 | 45.8 | 60.4 | 75.0 | 83.3 |
| **Convenience** | | | | | | | | | | | |
|  | Persistence | | |  |  |  |  |  |  |  |  |
|  |  | 6 months | |  |  |  |  |  |  |  |  |
|  |  |  | Total | 49 | 72.4 | 15.5 | 27.8 | 61.1 | 72.2 | 83.3 | 100.0 |
|  |  |  | Yes | 44 | 73.7 | 15.4 | 27.8 | 66.7 | 72.2 | 86.1 | 100.0 |
|  |  |  | No | 5 | 61.1 | 11.8 | 44.4 | 55.6 | 61.1 | 72.2 | 72.2 |
|  |  | 12 months | |  |  |  |  |  |  |  |  |
|  |  |  | Total | 49 | 72.4 | 15.5 | 27.8 | 61.1 | 72.2 | 83.3 | 100.0 |
|  |  |  | Yes | 40 | 74.4 | 13.7 | 44.4 | 66.7 | 72.2 | 86.1 | 100.0 |
|  |  |  | No | 9 | 63.6 | 20.2 | 27.8 | 55.6 | 66.7 | 72.2 | 94.4 |
|  | Adherence | | |  |  |  |  |  |  |  |  |
|  |  | 6 months | |  |  |  |  |  |  |  |  |
|  |  |  | Total | 49 | 72.4 | 15.5 | 27.8 | 61.1 | 72.2 | 83.3 | 100.0 |
|  |  |  | Yes | 39 | 74.9 | 14.7 | 27.8 | 66.7 | 72.2 | 88.9 | 100.0 |
|  |  |  | No | 10 | 62.8 | 15.3 | 44.4 | 50.0 | 63.9 | 72.2 | 94.4 |
|  |  | 12 months | |  |  |  |  |  |  |  |  |
|  |  |  | Total | 49 | 72.4 | 15.5 | 27.8 | 61.1 | 72.2 | 83.3 | 100.0 |
|  |  |  | Yes | 35 | 76.8 | 12.5 | 55.6 | 66.7 | 72.2 | 88.9 | 100.0 |
|  |  |  | No | 14 | 61.5 | 17.1 | 27.8 | 50.0 | 63.9 | 72.2 | 94.4 |
| **Global satisfaction** | | | | | | | | | | | |
|  | Persistence | | |  |  |  |  |  |  |  |  |
|  |  | 6 months | |  |  |  |  |  |  |  |  |
|  |  |  | Total | 49 | 76.7 | 17.0 | 16.7 | 66.7 | 83.3 | 91.7 | 100.0 |
|  |  |  | Yes | 44 | 78.2 | 14.3 | 33.3 | 66.7 | 83.3 | 91.7 | 100.0 |
|  |  |  | No | 5 | 63.3 | 32.1 | 16.7 | 50.0 | 66.7 | 83.3 | 100.0 |
|  |  | 12 months | |  |  |  |  |  |  |  |  |
|  |  |  | Total | 49 | 76.7 | 17.0 | 16.7 | 66.7 | 83.3 | 91.7 | 100.0 |
|  |  |  | Yes | 40 | 79.2 | 12.8 | 50.0 | 66.7 | 83.3 | 91.7 | 100.0 |
|  |  |  | No | 9 | 65.7 | 27.8 | 16.7 | 50.0 | 66.7 | 83.3 | 100.0 |
|  | Adherence | | |  |  |  |  |  |  |  |  |
|  |  | 6 months | |  |  |  |  |  |  |  |  |
|  |  |  | Total | 49 | 76.7 | 17.0 | 16.7 | 66.7 | 83.3 | 91.7 | 100.0 |
|  |  |  | Yes | 39 | 80.6 | 12.4 | 50.0 | 66.7 | 83.3 | 91.7 | 100.0 |
|  |  |  | No | 10 | 61.7 | 24.0 | 16.7 | 50.0 | 66.7 | 75.0 | 100.0 |
|  |  | 12 months | |  |  |  |  |  |  |  |  |
|  |  |  | Total | 49 | 76.7 | 17.0 | 16.7 | 66.7 | 83.3 | 91.7 | 100.0 |
|  |  |  | Yes | 35 | 81.0 | 12.6 | 50.0 | 66.7 | 83.3 | 91.7 | 100.0 |
|  |  |  | No | 14 | 66.1 | 22.0 | 16.7 | 58.3 | 66.7 | 83.3 | 100.0 |

^a^TSQM (version II) items 4, 5, and 6, on which the TSQM side effects domain score is based, are only to be answered by patients who experienced side effects (36 of the 49 patients who completed the TSQM [version II] at baseline). By definition, only one item may be missing in order to calculate the domain score; 7 patients answered only one of the items 4, 5, or 6 and were therefore excluded from this analysis.

Max: maximum; min: minimum; Q: quartile; TSQM: Treatment Satisfaction Questionnaire for Medication.
