# Supplementary material for: Ascertaining Medication Use and Patient-Reported Outcomes via an App and Exploring Gamification in Patients With Multiple Sclerosis Treated With Interferon β-1b: Observational Study
Source: JMIR Form Res. 2022 Mar 14;6(3):e31972. doi: 10.2196/31972 (PMC8929528; doi:10.2196/31972)
Supplement: Multimedia Appendix 8 [file formative_v6i3e31972_app8.doc]

## Multimedia Appendix

# Ascertaining Medication Use and Patient-Reported Outcomes Via an App and Exploring Gamification in Patients With Multiple Sclerosis Treated With Interferon *β*-1b: Observational Study

Volker Limmroth, MD; Kirsten Bayer-Gersmann, BEng; Christian Müller, PhD; Markus Schürks, MD, MSc

**Table.** Baseline satisfaction with the PSDMP, BETACONNECT, and app stratified by persistence and adherence at 6 months and 12 months

|  |  |  |  | **Baseline satisfaction with the PSDMP** | | | **Baseline satisfaction with the BETACONNECT** | | | **Baseline satisfaction with the app** | | |
| --- | --- | --- | --- | --- | --- | --- | --- | --- | --- | --- | --- | --- |
|  |  |  |  | **Total** | **‘Very satisfied’ OR ‘Satisfied’** | **‘Neither satisfied nor dissatisfied’ OR ‘Dissatisfied’ OR ‘Very dissatisfied’** | **Total** | **‘Very satisfied’ OR ‘Satisfied’** | **‘Neither satisfied nor dissatisfied’ OR ‘Dissatisfied’ OR ‘Very dissatisfied’** | **Total** | **‘Very satisfied’ OR ‘Satisfied’** | **‘Neither satisfied nor dissatisfied’ OR ‘Dissatisfied’ OR ‘Very dissatisfied’** |
| Persistence | | |  |  |  |  |  |  |  |  |  |  |
|  | 6 months | |  |  |  |  |  |  |  |  |  |  |
|  |  | Total | n | 49 | 34 | 15 | 49 | 25 | 24 | 49 | 39 | 10 |
|  |  |  | % | 100.0 | 69.4 | 30.6 | 100.0 | 51.0 | 49.0 | 100.0 | 79.6 | 20.4 |
|  |  | Yes | n | 44 | 32 | 12 | 44 | 23 | 21 | 44 | 36 | 8 |
|  |  |  | % | 100.0 | 72.7 | 27.3 | 100.0 | 52.3 | 47.7 | 100.0 | 81.8 | 18.2 |
|  |  | No | n | 5 | 2 | 3 | 5 | 2 | 3 | 5 | 3 | 2 |
|  |  |  | % | 100.0 | 40.0 | 60.0 | 100.0 | 40.0 | 60.0 | 100.0 | 60.0 | 40.0 |
|  | 12 months | |  |  |  |  |  |  |  |  |  |  |
|  |  | Total | n | 49 | 34 | 15 | 49 | 25 | 24 | 49 | 39 | 10 |
|  |  |  | % | 100.0 | 69.4 | 30.6 | 100.0 | 51.0 | 49.0 | 100.0 | 79.6 | 20.4 |
|  |  | Yes | n | 40 | 29 | 11 | 40 | 21 | 19 | 40 | 33 | 7 |
|  |  |  | % | 100.0 | 72.5 | 27.5 | 100.0 | 52.5 | 47.5 | 100.0 | 82.5 | 17.5 |
|  |  | No | n | 9 | 5 | 4 | 9 | 4 | 5 | 9 | 6 | 3 |
|  |  |  | % | 100.0 | 55.6 | 44.4 | 100.0 | 44.4 | 55.6 | 100.0 | 66.7 | 33.3 |
| Adherence | | |  |  |  |  |  |  |  |  |  |  |
|  | 6 months | |  |  |  |  |  |  |  |  |  |  |
|  |  | Total | n | 49 | 34 | 15 | 49 | 25 | 24 | 49 | 39 | 10 |
|  |  |  | % | 100.0 | 69.4 | 30.6 | 100.0 | 51.0 | 49.0 | 100.0 | 79.6 | 20.4 |
|  |  | Yes | n | 39 | 27 | 12 | 39 | 21 | 18 | 39 | 32 | 7 |
|  |  |  | % | 100.0 | 69.2 | 30.8 | 100.0 | 53.8 | 46.2 | 100.0 | 82.1 | 17.9 |
|  |  | No | n | 10 | 7 | 3 | 10 | 4 | 6 | 10 | 7 | 3 |
|  |  |  | % | 100.0 | 70.0 | 30.0 | 100.0 | 40.0 | 60.0 | 100.0 | 70.0 | 30.0 |
|  | 12 months | |  |  |  |  |  |  |  |  |  |  |
|  |  | Total | n | 49 | 34 | 15 | 49 | 25 | 24 | 49 | 39 | 10 |
|  |  |  | % | 100.0 | 69.4 | 30.6 | 100.0 | 51.0 | 49.0 | 100.0 | 79.6 | 20.4 |
|  |  | Yes | n | 35 | 24 | 11 | 35 | 19 | 16 | 35 | 29 | 6 |
|  |  |  | % | 100.0 | 68.6 | 31.4 | 100.0 | 54.3 | 45.7 | 100.0 | 82.9 | 17.1 |
|  |  | No | n | 14 | 10 | 4 | 14 | 6 | 8 | 14 | 10 | 4 |
|  |  |  | % | 100.0 | 71.4 | 28.6 | 100.0 | 42.9 | 57.1 | 100.0 | 71.4 | 28.6 |

PSDMP: Patient Support and Disease Management Program.
