# Supplementary figures and images for: Correction: Ascertaining Medication Use and Patient-Reported Outcomes via an App and Exploring Gamification in Patients With Multiple Sclerosis Treated With Interferon β-1b: Observational Study
Source: JMIR Form Res. 2022 Mar 17;6(3):e38002. doi: 10.2196/38002 (PMC8972101; doi:10.2196/38002)

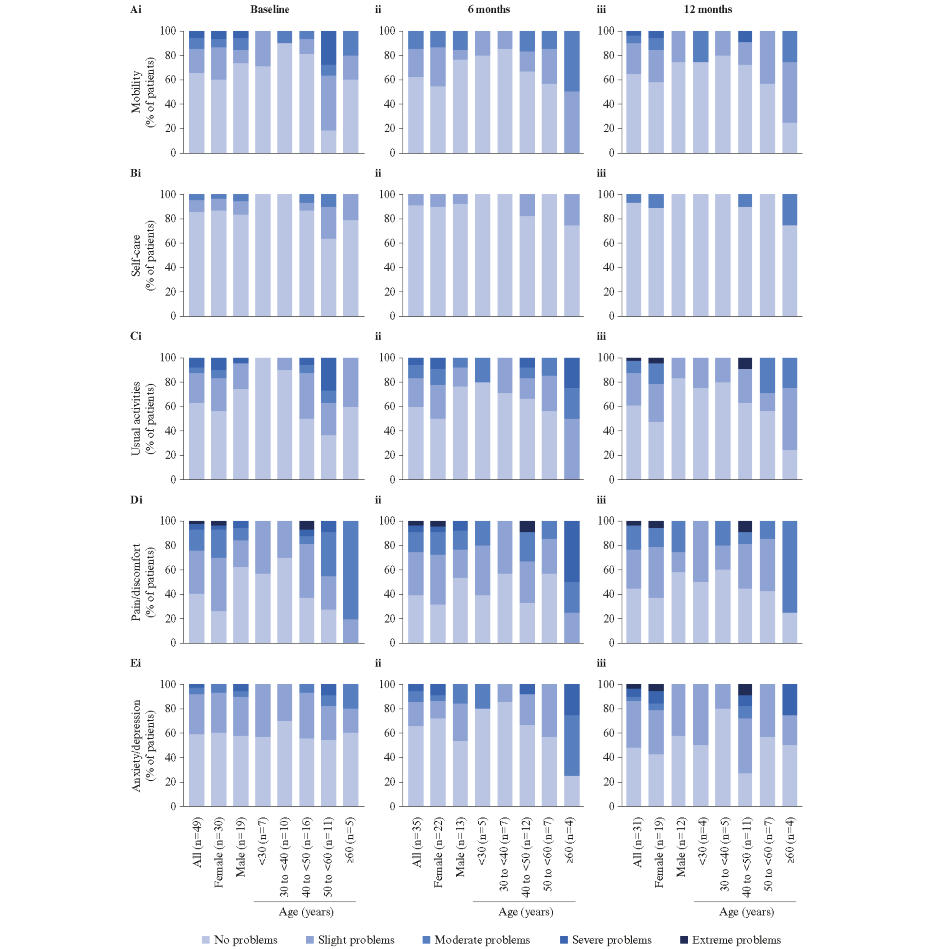

Supplement: Multimedia Appendix 1 [file formative_v6i3e38002_app1.png]
